# Graph neural networks-based prediction of drug gene association of P2X receptors in periodontal pain

**DOI:** 10.1016/j.jobcr.2024.04.008

**Published:** 2024-04-21

**Authors:** Pradeep Kumar Yadalam, Prabhu Manickam Natarajan, Seyed Ali Mosaddad, Artak Heboyan

**Affiliations:** a Department of Periodontics, Saveetha Dental College, Saveetha Institute of Medical and Technical Sciences, Saveetha University, Chennai, India; b Department of Clinical Sciences, Centre of Medical and Bio-allied Health Sciences and Research, College of Dentistry, Ajman University, Ajman, United Arab Emirates; c Student Research Committee, School of Dentistry, Shiraz University of Medical Sciences, Shiraz, Iran; d Department of Prosthodontics, Faculty of Stomatology, Yerevan State Medical University after Mkhitar Heratsi, Yerevan, Armenia; e Department of Prosthodontics, School of Dentistry, Tehran University of Medical Sciences, Tehran, Iran

**Keywords:** Drug, Genes, Purinergic P2X receptor antagonists, Neural network, Pain, Periodontal diseases

## Abstract

The P2X7 receptor, a member of the P2X receptor family, plays a crucial role in various physiological processes, particularly pain perception. Its expression across immune, neuronal, and glial cells facilitates the release of pro-inflammatory molecules, thereby influencing pain development and maintenance, as evidenced by its association with pulpitis in rats. Notably, P2X receptors such as P2X3 and P2X7 are pivotal in dental pain pathways, making them promising targets for novel analgesic interventions. Leveraging graph neural networks (GNNs) presents an innovative approach to model graph data, aiding in the identification of drug targets and prediction of their efficacy, complementing advancements in genomics and proteomics for therapeutic development. In this study, 921 drug-gene interactions involving P2X receptors were accessed through https://www.probes-drugs.org/. These interactions underwent meticulous annotation, preprocessing, and subsequent utilization to train and assess GNNs. Furthermore, leveraging Cytoscape, the CytoHubba plugin, and other bioinformatics tools, gene expression networks were constructed to pinpoint hub genes within these interactions. Through analysis, SLC6A3, SLC6A2, FGF1, GRK2, and PLA2G2A were identified as central hub genes within the context of P2X receptor-mediated drug-gene interactions. Despite achieving a 65 percent accuracy rate, the GNN model demonstrated suboptimal predictive power for gene-drug interactions associated with oral pain. Hence, further refinements and enhancements are imperative to unlock its full potential in elucidating and targeting pathways underlying oral pain mechanisms.

## 1 Introduction

Periodontal pain is a complex experience involving various functions and sensations, and both acute and chronic pains can decrease the quality of life and the ability to work.^1^, ^2^, ^3^, ^4^ Roughly one in four adults globally suffer from pain in the oral and maxillofacial regions, with intense inflammation of the dental pulp being the primary reason for this discomfort. Adenosine 5′-triphosphate (ATP) functions as an excitatory neurotransmitter that plays a crucial role in the transmission of signals between cells and the development of pain. The P2X7 receptor, belonging to the ATP receptor family, can be found across a range of cells and is instrumental in managing pathological pain 5, 6, 7. Studies have shown the upregulation of P2X2, P2X3, P2X5, and P2X7 receptors concerning dental pain in rats and humans.^8^, ^9^, ^10^

Purinergic signaling pathways in the tooth affect cytokine expression and secretion. ATP release stimulates osteopontin and receptor activator of nuclear factor kappa beta (NF-κB ligand) (RANKL), while P2X3 and P2X2/P2X3 receptors transmit nociceptive signals.^11^^,^^12^ LL-37, an antimicrobial peptide, up-regulates IL-8, which is disrupted in periodontitis in gingival fibroblasts.^5^^,^^13^ Blocking LL-37 may link inflammation and purinergic signaling. The P2Y 12 antagonist clopidogrel reduces IL-6 and TNF-α levels in periodontal disease.^14^^,^^15^

Research indicates that the P2X7 receptor plays a critical role in the development of inflammation and pain in rat pulpitis. The blockade of the P2X7 receptor with the antagonist A-740003 has been shown to alleviate inflammation and mitigate pain in rat models suffering from pulpitis. Additionally, P2X receptors, including the P2X3 subtype, are acknowledged for their involvement in dental discomfort. Consequently, antagonists targeting these receptors are being highlighted as vital focal points for the innovation of new pain-relief drugs.^16^ The P2X7 receptor is also linked to inflammation-associated impairment of periodontal ligament stem cells (PDLSCs).

P2X receptors, ATP-gated ion channels, are involved in transmitting pain signals in dental and periodontal conditions. Purines transmit pain from the oral cavity, with low ATP concentrations initiating action potentials in dental pulp nociceptive neurons. These neurons, activated by ATP, link purinergic signaling and the P2X 3 receptor to pain. Mechanical distortion of odontoblasts triggers ATP release and pain sensation via P2X3 receptors in human nerve fibers and dental pulp.^13^

The P2X 3 receptor is crucial for tooth movement pain and may be a target for pain control in tooth movement.^17^ Targeting these P2X receptors is vital for targeting oral pain. Identifying drug-gene associations is essential for novel drug identification and can cure severe pain. In order to effectively address oral pain, it is important to target P2X receptors, as they play a significant role in pain transmission. By focusing on these receptors, potential treatments for oral pain can be developed. Additionally, identifying associations between specific drugs and genes is crucial for discovering novel medicines that can effectively relieve severe pain 2,18.

Identifying gene-drug associations is crucial for understanding regulatory networks and developing precision drugs. Computational methods are needed to reduce time and cost, especially with high-throughput datasets.^19^^,^^20^ Recent studies show that gene-drug associations are many-to-many, allowing for the identification of gene-drug modules. Efforts have been made to develop computational methods for identifying many-to-many associations in biological datasets, such as data integration frameworks, joint matrix factorization, and regression methods. Graph neural networks (GNNs) model graph data using techniques originally designed for imaging, allowing for fixed-length vector representation and analysis of nodes of interest, such as genes.^18^^,^^21^, ^22^, ^23^ Neural networks play a role in understanding and predicting dental or periodontal pain by analyzing complex patterns and relationships in data related to pain symptoms, genetic factors, and drug interactions.^23^ Researchers are developing personalized treatment options for dental and periodontal conditions by tracking gene-drug interactions based on individual genetic and drug responses.^24^ GNN models can uncover hidden patterns in gene-drug interaction networks, aiding drug target identification and effectiveness prediction and collaborating with genomics and proteomics for new therapeutic interventions. This study aims to graph neural networks-based prediction of drug-gene association of P2X receptors in periodontal pain.

## 2 Methods

Using https://www.probes-drugs.org/,^25^ 921 drug-gene interactions of - P2X receptors with drug signaling were downloaded, the drug-protein gene data set was annotated and preprocessed, and outliers were removed for protein interactions. This drug-gene annotated dataset contains the ID name, biochemical activity, mode of action, and drug-gene interaction type like protein-coding and Chimeric Protein with 80 percent training and 20 percent testing, with mode of action as a target label for graph neural networks.

## 3 Network analysis and hub gene identification

Cytoscape,^26^ along with the CytoHubba plugin and various bioinformatics tools, enables the creation of gene expression networks for identifying hub genes. Using topological analysis methods provided by CytoHubba, nodes in the protein-protein interaction (PPI) network can be ranked based on network connectivity.

## 4 Graph neural network-architecture

Graph Neural Network^19^, ^20^, ^21^ architecture is a neural network operating on graph-structured data.

The basic architecture of a GNN consists of the following components.

1. Representation of Graph: The given data is showcased in the form of a graph. In this graphical representation, drugs and genes are depicted as nodes, while the connections between them are illustrated through edges. Each node is often accompanied by certain features or traits.
2. Embedding of Nodes: The preliminary action involves embedding every node within the graph into a vector space of reduced dimensionality. This embedding is accomplished by applying a transformation to the node features via an embedding layer that can be adjusted.
3. Messages passing: At the heart of a Graph Neural Network (GNN) lies the message exchange procedure, whereby nodes communicate information amongst themselves. By gathering and refreshing information from its neighbors, each node learns a revamped representation. This exchange is cyclically done through several layers dedicated to message passing.
4. Pooling of Graphs: Occasionally, it is vital to compile information from the entire graph to derive an all-encompassing representation. To achieve this, graph pooling methods such as convolutional pooling or attention mechanisms are utilized to generate an embedding that reflects the entire graph.
5. Output layer: The concluding layer(s) within a GNN serve the purpose of making predictions or executing tasks downstream, like classifying nodes, predicting links, or regressing graphs. Hyperparameter tuning includes Activation function: leaky RELU, LEARNING RATE-0.001, Dropout: 0.2, BATCH Size: 100, REGULARISATION-L2.

## 5 Results

After analysis, these drug-gene interactions in P2X receptors showed 921 datasets with top hub genes, revealing SLC6A3, SLC6A2, FGF1, GRK2, and PLA2G2A.

The network analysis shows 491 nodes and 921 edges in the drug-gene interaction network. Each node represents a gene or drug, and the average number of neighbors is 3658. This indicates high connectivity, with each node connected to approximately 3658 other nodes. The analysis can help identify important nodes and their relationships, potentially revealing their complexity and functional implications.

Graph neural networks analysis of protein-drug interactions reveals an accuracy of 65 percent, f1 score-0.7921, precision −0.72, recall −0.8696, and Area Under the Curve (AUC)-0.5912.

However, the accuracy and AUC suggest room for improvement. The model's ability to discriminate between positive and negative instances is moderate but relatively low. The F1 score, precision, and recall show good performance, but the AUC indicates room for improvement. Further analysis and optimization may be required to enhance the model's predictive capabilities. Overall, the graph neural network analysis of protein-drug interactions shows potential for improvement.

The Receiver Operating Characteristic (ROC) curve measures the trade-off between true positive rate (TPR) and false positive rate (FPR) in drug-gene interactions. The GNN model's AUC value of 0.5912 indicates moderate discriminatory power but falls short of high accuracy and precision.

## 6 Discussion

Top hub genes include FGF-1, which activates P2X7 receptors and induces ATP release from spinal astrocytes, revealing the pain mechanism. GRK2 phosphorylates the P2X receptor, making the receptor less responsive to ATP, called desensitization.^6^, ^7^ Desensitization is important for regulating the activity of P2X receptors and preventing them from becoming overstimulated in oral facial pain.^8^,^10^,^15^ PLA2G2A may be activated by P2X receptor-mediated calcium influx, leading to the production of arachidonic acid and its downstream metabolites.^13^ These metabolites could, in turn, modulate P2X receptor function in periodontal pain.^27^, ^28^, ^29^ Hub genes involved in drugs are highly connected genes in the gene-drug interaction network. These genes are important because they often have a central role in the mechanism of action of drugs and their targets. Researchers can gain insights into the molecular pathways and biological processes underlying drug response and efficacy by studying these hub genes.^30^^,^^31^

Graph neural network (GNN) modeling is a machine learning technique that analyzes complex relationships in graph-structured data, such as gene-drug interaction networks. When combined with genomics and proteomics, it helps researchers identify hidden patterns, predict drug effectiveness, and discover new therapeutic interventions for diseases like oral pain 32. These hub genes involved in drugs could play an important role in drug targets, and graph neural network modeling will help identify and predict novel drugs in P2X receptors.^33^

**Fig. 1:**
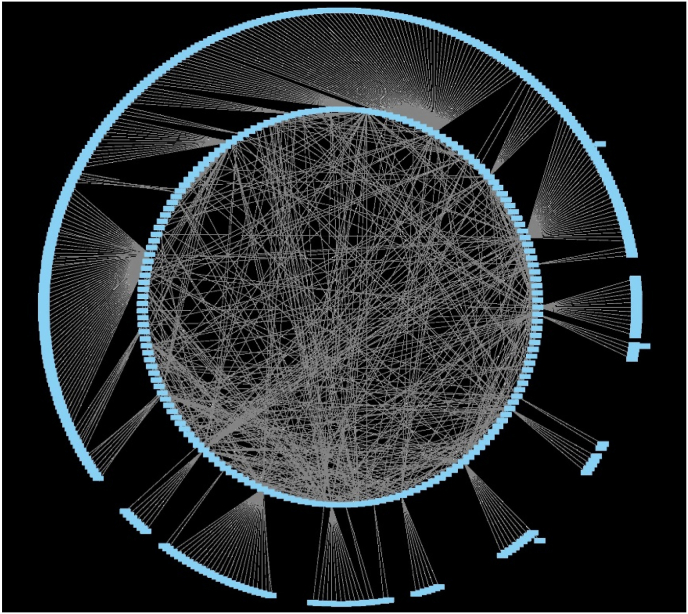
It shows NODES-491, EDGES -921, AVERAGE NO OF NEIGHBORS – 3658.

**Fig. 2:**
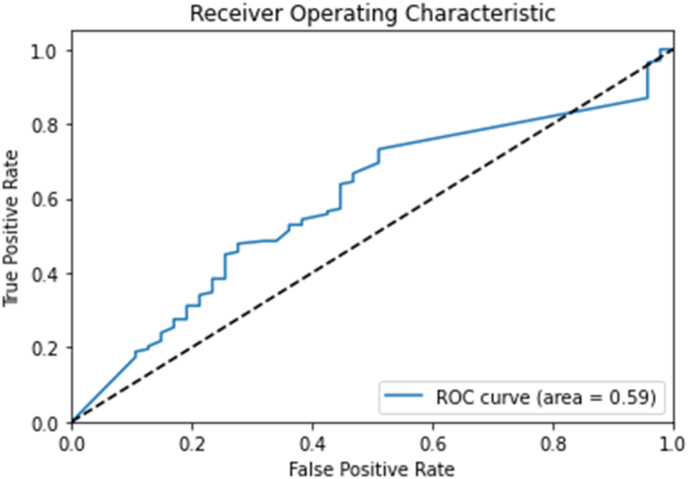
It shows the acceptable accuracy of the GNN model in drug-gene interactions.

**Fig. 3:**
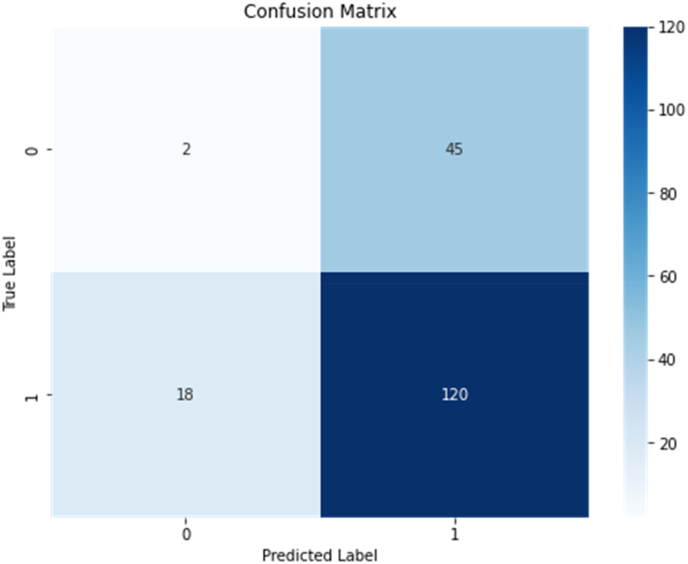
It shows a confusion matrix of all classes of protein–gene interactions.

Accurate computational approaches are needed to predict drug-target interactions and their effects, which may require additional validation steps.^3^^,^^9^^,^^14^^,^^19^, ^20^, ^21^^,^^33^^,^^34^ The graph neural network analysis model has moderate accuracy but high precision, recall, and F1 score. It correctly predicted the interaction between proteins and drugs in 65 percent of cases (Fig. 1, Fig. 2, Fig. 3). However, the model's ability to distinguish between positive and negative interactions may be poor. The AUC value of 0.5912 indicates that the model's performance on the ROC curve is poor, suggesting further improvements and optimizations may be required to enhance its performance. In the context of translational value for P2X receptors in periodontal pain, these results provide some insights into the potential interactions between proteins and drugs in this specific scenario. The moderate accuracy suggests that the model can make correct predictions in most cases, but there is still room for improvement.

The model accurately identifies positive and negative interactions between proteins and drugs but struggles to distinguish between positive and negative interactions, potentially leading to false positives or negatives in identifying potential therapeutic targets or drug candidates for P2X receptors in periodontal pain. To enhance translational value, further improvements and optimizations, such as refining the model's architecture, incorporating additional features, or adjusting training parameters, are needed.

## 7 Conclusion

The model's ability to accurately predict gene-drug interactions in the network is suboptimal. Therefore, further refinements and enhancements are needed to improve the model's predictive power and unlock its full potential in discovering drug-target interactions for oral pain by targeting P2X receptors.

## Funding

There was no funding provided.

## Availability of data and material

Not applicable.

## Ethical approval and consent to participate

Not applicable.

## Authors' contributions

Conceptualization, Methodology, Software, Validation, Formal analysis, Investigation, Resources, Data Curation, Writing - Original Draft, Writing - Review and editing, Visualization, Supervision, and Project administration: P.K.Y., P.M.N., S.A.M., and A.H. All authors have read and agreed on the current version of the manuscript to be published.

## Consent to participate

Not applicable.

## Consent for publication

Not applicable.

**Table:** Table of the abbreviations used in the study.

Number	Abbreviations	Full form
1	P2X	ATP-gated P2X receptor cation channel family
2	ATP	Adenosine Tri Phosphate
3	RANKL	Receptor activator of nuclear factor kappa beta ligand
4	GNN	Graph neural networks
5	PPI	Protein-protein interactions
6	PDLSCs	Periodontal ligament stem cells
7	AUC	Area Under the Curve
8	ROC	Receiver Operating Characteristic
9	TPR	True positive rate
10	FPR	False positive rate

## Declaration of competing interest

The authors claim to have no conflicts of interest.
